# An MRI-Derived Definition of MCI-to-AD Conversion for Long-Term, Automatic Prognosis of MCI Patients

**DOI:** 10.1371/journal.pone.0025074

**Published:** 2011-10-12

**Authors:** Yaman Aksu, David J. Miller, George Kesidis, Don C. Bigler, Qing X. Yang

**Affiliations:** 1 Center for NMR Research, Department of Radiology, Penn State University College of Medicine, Hershey, Pennsylvania, United States of America; 2 Electrical Engineering Department, Penn State University, University Park, Pennsylvania, United States of America; 3 Computer Science and Engineering Department, Penn State University, University Park, Pennsylvania, United States of America; 4 Center for Emerging Neurotechnology and Imaging, Penn State University College of Medicine, Hershey, Pennsylvania, United States of America; Biological Research Center of the Hungarian Academy of Sciences, Hungary

## Abstract

Alzheimer's disease (AD) and mild cognitive impairment (MCI) are of great current research interest. While there is no consensus on whether MCIs actually “convert” to AD, this *concept* is widely applied. Thus, the more important question is not *whether* MCIs convert, but *what is the best such definition*. We focus on automatic prognostication, nominally using only a baseline brain image, of whether an MCI will convert within a multi-year period following the initial clinical visit. This is not a traditional supervised learning problem since, in ADNI, *there are no definitive labeled conversion examples*. It is not unsupervised, either, since there are (labeled) ADs and Controls, as well as cognitive scores for MCIs. Prior works have defined MCI subclasses based on whether or not clinical scores significantly change from baseline. There are concerns with these definitions, however, since, e.g., most MCIs (*and ADs*) do not change from a baseline CDR = 0.5 at any subsequent visit in ADNI, even while physiological changes may be occurring. These works ignore rich *phenotypical* information in an MCI patient's brain scan and labeled AD and Control examples, in defining conversion. We propose an innovative definition, wherein an MCI is a converter if any of the patient's brain scans are classified “AD” by a Control-AD classifier. This definition *bootstraps* design of a *second* classifier, *specifically trained to predict whether or not MCIs will convert*. We thus predict whether an AD-Control classifier will predict that a patient has AD. Our results demonstrate that this definition leads not only to much higher prognostic accuracy than by-CDR conversion, but also to subpopulations more consistent with *known* AD biomarkers (including CSF markers). We also identify key *prognostic* brain region biomarkers.

## Introduction

The dementing illness Alzheimer's disease (AD), and the transitional state between normal aging and AD referred to as mild cognitive impairment (MCI) continue to be widely studied. Individuals diagnosed with MCI have memory impairment, yet without meeting dementia criteria. Annually
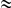
10–15% of people with MCI are diagnosed with AD [1]. Moreover, prior to symptom onset, brain abnormalities have been found in people with MCI, as ascertained by retroactive evaluation of longitudinal MRI scans [2]. There is no consensus on whether MCI patients actually “convert” to AD. First, some MCI patients may suffer from other neurodegenerative disorders (e.g., Lewy body dementia, vascular dementia and/or frontotemporal dementia). Second, it is possible that all other MCI patients already have AD, but at a preclinical stage. AD diagnosis itself may not be considered definitive without e.g. confirming pathologies such as the amyloid deposits detectable at autopsy. Regardless of whether MCI patients truly “convert” to AD or not, the concept of MCI-to-AD conversion has been widely applied, e.g. [3], [4], [5], [6], [7] and is utilitarian – defining MCI (converter and nonconverter) subgroups allows use of statistical group difference tests and machine learning methods to help identify early disease biomarkers and to build models for predicting disease progression. For these purposes, the more important question is not whether MCIs convert, but rather *what is the best such definition*.

Accordingly, here we focus on the following **Aim**: automatic prognostication, (nominally) using only a baseline brain scan, of whether an MCI individual will convert to AD within a multi-year (three year) period following an initial (baseline) clinical visit. Our system performs three-year ahead prediction because it is designed based on the ADNI database, which followed participants for a period of up to three years. (Data used in preparation of this article were obtained from the Alzheimer's Disease Neuroimaging Initiative (ADNI) database adni.loni.ucla.edu. As such, the investigators within the ADNI contributed to the design and implementation of ADNI and/or provided data but did not participate in analysis or writing of this report. A complete listing of ADNI investigators can be found at: http://adni.loni.ucla.edu/wp-content/uploads/how_to_apply/ADNI_Authorship_List.pdf).

While only image voxel-based features are evaluated here for use by our classifier, our framework is extensible to incorporating other baseline visit clinical information (e.g. weight, gender, education level, genetic information, and clinical cognitive scores such as the Mini Mental State Exam (MMSE)) into the decisionmaking. Moreover, our approach can also incorporate the recent, promising cerebrospinal fluid (CSF) based markers [8]. However, as this requires an invasive spinal tap, we focus here on image scans, which are routinely performed for subjects with MCI.

We do not hypothesize that, within ADNI, there are actually two subclasses of MCI subjects when evaluated over the very long term – those that (eventually) convert to AD, and those that do not. Even if an overwhelming majority of MCI subjects will *eventually* convert, identifying the subgroup likely to convert *within several years* has several compelling utilities: 1) early prognosis, to assist family planning; 2) facilitating group-targeted treatments/drug trials; 3) we identify key *prognostic* brain “biomarker” regions, *i.e.* those found to be most critical for accurately discriminating our “converter” and “nonconverter” groups. These regions may shed light on disease etiology.

Distinguishing AD converters from nonconverters is a binary (two-class) classification problem. Moreover, it may appear this classification problem can be directly addressed via supervised learning methods [9]. However, it is in fact an *unconventional* problem, lying somewhere *between* supervised classification and unsupervised classification (clustering), and thus requiring a unique approach. To appreciate this, consider the ADNI cohort of MCI individuals. ADNI consists of clinical information and image scans on hundreds of participants, taken at six-month intervals for up to three years. A clinical label (AD, MCI, or Control) was assigned to each participant at first visit. Clinicians derive the AD/MCI/Control label based on multiple criteria, which may include Clinical Dementia Rating (CDR), whose possible values are: *0 = none, 0.5 = questionable, 1 = mild, 2 = moderate, 3 = severe*. Even though a *probable AD* definition based on CDR and MMSE scores and NINCDS/ADRDA criteria has been used (e.g. [10], [7]) to provide follow-up assessment for MCI patients, this is strictly a clinically driven definition, based on a clinical rating (CDR) and a cognitive score (MMSE) whose difficulties will be pointed out shortly. This is not a definitive (autopsy-based) determination of AD, nor is it a definition based on physiological brain changes. Even if the *probable AD* definition has very high *specificity*, it may not be sufficiently sensitive, i.e. there may be patients who are undergoing significant physiological brain changes consistent with conversion, yet without clinical manifestation.

Accordingly, we will approach the conversion problem from a perspective as agnostic and unbiased as possible, and simply state that it is not definitively known which MCI participants in ADNI truly converted to AD within three years. In conventional supervised classifier learning, one has labeled training examples, used for designing the classifier, and labeled test examples, used to estimate the classifier's generalization accuracy. For predicting whether MCI participants in ADNI convert to AD, we in fact have neither. Thus, our problem is not conventional supervised learning. On the other hand, consider *unsupervised* clustering [9]. Here, even if one knows the number of clusters (classes) present, there is no prior knowledge on what is a good clustering – one is simply looking for underlying grouping tendency in the data. Clearly, our problem does not fit unsupervised clustering, either – while we have no ground-truth labeled MCI converter/nonconverter instances per se, 1) there are two designated classes of interest (converter and nonconverter); and 2) there are known class characteristics – conversion to AD should, plausibly, mean that: (i) a clinical measure such as CDR or a cognitive measure has changed *and/or* (ii) there are changes in brain region features or in CSF biomarkers more characteristic of AD subjects than normal/healthy subjects. Note that in ADNI we do have plentiful labeled AD and normal/healthy (Control) examples to help assess ii).

Based on the above, MCI prognosis is an interesting and novel problem, lying somewhere between supervised and unsupervised classification. The crux of this problem is to craft criteria through which meaningful MCI subgroups can be defined, well-capturing notions of “AD converter” and “nonconverter”. To help guide development and evaluation of candidate definitions, we state the following three **desiderata**: 1) The proposed definition of AD converter should be plausible and should exploit the available, relevant information in the ADNI database (e.g. image data, labeled AD and Control examples, and clinical information). To appreciate 1), note that the MCI population could be dichotomized in *many* ways, *e.g.* by height, and there might be significant clustering tendency with respect to height, but such a grouping is likely meaningless for MCI prognosis; 2) A classifier trained based on these class definitions should *generalize* well on test data (not used for training the classifier) – this quantifies how accurately we can discriminate the classes that we have defined. Equivalently, it tells us whether the *features* we are using are adequate for well-discriminating the classes we define. If we create what we believe to be good definitions, but ones that cannot be accurately discriminated, that would not be useful clinically; 3) The class definitions should be validated using known AD conversion biomarkers ( *i.e.*, external measures) such as measured changes from baseline in volumes or final visit volumes of brain regions known to be associated with the disease [11], CSF biomarkers [12], and cognitive test scores (such as the clinical MMSE measure).

### Prior Related Work

Several prior works, e.g. [4], [5], [13], defined converter and nonconverter classes *solely* according to whether the baseline visit CDR score of 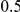 rose or stayed the same over all visits. Change in CDR has also been used as surrogate ground-truth for cognitive decline in a number of other papers, e.g. [14], [6]. While CDR gives a workable conversion definition, it should be evaluated with respect to the three desiderata above. We will evaluate 2) and 3) in the sequel. With respect to 1), one should challenge a CDR-based conversion definition. First, CDR is not an effective discriminator between the AD and MCI groups, *i.e.* there is *very significant* AD-MCI overlap, not only with respect to CDR = 1 *but even 0.5* – for ADNI, the majority of the hundreds of AD subjects used in our experiments start (at first visit) at CDR = 0.5 and stay at 0.5 at *all* later visits; likewise, nearly all MCI subjects start at 0.5, with a large majority of these *also* staying at 0.5 for all visits. This latter fact further implies difficulties in finding an adequate number of conversion-by-CDR subjects in ADNI, both for accurate classifier training and test set evaluation. For the even more stringent *probable AD* definition (meeting MMSE and NINCDS/ADRDA criteria, in addition to CDR changing from 0.5 to 1) there are necessarily even *fewer* MCI converters for classifier training and testing. Second, a purely CDR-based (or “probable AD” based) conversion definition ignores the (rich) *phenotypical* information in an MCI subject's image brain scans and does not exploit the labeled AD and normal/healthy (Control) examples in ADNI. These prior works do treat features derived from brain scans as the *covariates* (the inputs) used by the classifier/predictor. However, we believe the MCI brain scans can themselves be used, in conjunction with the labeled AD and Control examples, to help define more accurate surrogate ground-truth.

Previous work has demonstrated that structural MRI analysis is useful for identifying AD biomarkers in individual brain regions [15], [16], [17] – *e.g.*, cortical thinning [18], [19], ventricle dilation and gaping [14], [3], [20], volumetric and shape changes in the hippocampus and entorhinal cortex [21], [22], [23], and temporal lobe shrinkage [24]. It is important to capture interaction effects across multiple brain regions [2], [5], [25], [13]. did jointly analyze voxels (or regions) spanning the entire brain and did build classifiers or predictors. Moreover, as part of their work [13], investigated prediction of future decline in MCI subjects working from baseline MRI scans, which is the primary subject of our current paper. However, there are several limitations of these past works. First, all these studies used the previously discussed CDR and cognitive measures such as MMSE, which has been described as noisy and unreliable, as the ground-truth prediction targets for classifier/regressor training. In [14], the authors state: “Cognitive assessments are notoriously variable over time, and there is increasing evidence that neuroimaging may provide accurate, reproducible measures of brain atrophy.” Even in [13], where MMSE was treated as the measure of decline and the ground-truth regression target, the authors acknowledged that “individual cognitive evaluations are known to be extremely unstable and depend on a number of factors unrelated to…brain pathology.” Such factors include sleep deprivation, depression, other medical conditions, and medications. Even though MMSE is widely used by clinicians, these comments (even if not universally accepted), do indicate MMSE by itself may not be so reliable in quantifying the disease state. Moreover, while [13] did build predictors of future MMSE scores working from baseline scans, this was not a main focus of their paper – their paper focused on predicting the current score. Their prognostic experiments involved a very small sample size (just 26 participants from the ADNI database). Accordingly, it is difficult to draw definitive conclusions about the accuracy of their prognostic model and their associated brain biomarkers. The main reason the authors chose such a small sample was, as the authors state: “A large part of…ADNI…are from patients who did not display significant cognitive decline…[these] would overwhelm the regression algorithm if..used in the…experiment.” While this statement (with cognitive decline measured according to MMSE) may be true, that does not mean many of those excluded ADNI subjects are not experiencing significant physiological brain changes/atrophy. The novel approach we next sketch is well-suited to identifying MCI subjects undergoing such changes.

### Our Neuroimaging-Driven, Trajectory-Based Approach

Here, we propose a novel approach for prognosticating putative conversion to AD driven by image-based information (and exploiting AD-Control examples), rather than by a single, non-image-based, weakly discriminating clinical measurement such as CDR. Our solution strategy is as follows. We first build an accurate image-based Control-AD classifier ( *i.e.*, using AD and Control subjects, we build a Support Vector Machine (SVM) classifier) (Vapnik, 1998). We then apply this classifier to a *training population* of MCI subjects – separately, for each subject visit, we determine whether the subject's image is on the AD side or the Control side of the SVM's fixed (hyperplane) decision boundary. In addition to a binary decision, the SVM gives a “score” – essentially the distance to the classifier's decision boundary. Thus, for each MCI subject, as a function of visits, we get an image-based “phenotypical” score trajectory. We fit a line to each subject's trajectory and extend the line to the sixth visit if the sixth visit is missing. We can then give the following *trajectory-based conversion definition*: if the extended line either starts on the AD side or crosses to the AD side over the six visits, we declare this person a “converter-by-trajectory”. Otherwise, this person is a “nonconverter-by-trajectory”. (A very small percentage of the MCI population, in our experiments often 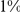 and not exceeding 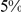, may unexpectedly start on the AD side and cross to the Control side. We treat these individuals as outliers and omit them from our experiments.) In this fashion, we *derive* ground-truth “converter” and “nonconverter” labels for an (initially unlabeled) training MCI population. These (now) labeled training samples *bootstrap* the design of a *second* SVM classifier which uses only the first-visit training set MCI images and is trained to predict whether or not an MCI patient is a “converter-by-trajectory”. Essentially, this second (prognostic) classifier is predicting whether, within three years, an AD-Control classifier will predict that a patient has AD. Via these two classifier design steps, we thus build a classification system for our (unconventional) pattern recognition task.

SVMs are widely used classifiers whose accuracy is attributed to their maximization of the “margin”, *i.e.* the smallest distance from any training point to the classification boundary. Since the SVM finds a linear discriminant function that *maximizes* margin, a significant change in score is generally needed to cross from the control side to the AD side, which is thus suggestive of conversion from MCI to AD. This is the premise underlying our approach.

The main contributions of our work are: 1) a novel machine learning framework for prognostication falling somewhere between traditional supervised and unsupervised learning; 2) a novel image-based prognosticator of MCI-to-AD conversion that we will demonstrate to achieve both better generalization accuracy and much higher correlation with known brain region biomarkers and with CSF-based markers than the CDR-based approach; 3) Identification of the brain regions most critical for accurately discriminating between our “converter” and “nonconverter” groups, via application of margin-based feature selection (MFE) [26] to brain image classification, and demonstration of MFE's better performance than the well-known RFE method [27] on this domain.

## Methods

### 2.1 Subjects and MRI data

We used 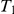-weighted ADNI images (data used in the preparation of this article were obtained from the Alzheimer's Disease Neuroimaging Initiative (ADNI) database (adni.loni.ucla.edu)) that have undergone image correction described at the ADNI website. ADNI image correction steps include Gradwarp, N3, and scaling for gradient drift – see www.loni.ucla.edu/ADNI/Data/ADNI_Data.shtml. ADNI aims to recruit and follow 800 research participants in the 55–90 age range: approximately 200 elderly Controls, 400 people with MCI, and 200 people with AD. The number of Control, MCI, and AD participants in our analysis were 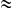
180, 300, and 120, respectively – experiment-specific detailed descriptions will be provided in Sec. 3. We processed the 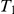-weighted images as described in the supplemental Document S1, producing new images from which we then obtained the features (next discussed) used by our statistical classifiers.

### 2.2 Features for classification

We chose as features the voxel intensities of a processed RAVENS image (we describe our processing of RAVENS images in the supplemental Document S1), a type of “volumetric density” image [28], [29], [30], [31] that has been validated for voxel-based analysis [29] and applied both to AD e.g. [4], [5], [13] and other studies e.g. [32]. Of particular interest [29], supported that voxel-based SPM statistical analysis, which we perform herein for comparison with our methods, can be performed on RAVENS images. For each of the three processed RAVENS tissue maps (gray matter (GM), white matter (WM), and ventricle), to reduce complexity for subsequent processing, we obtained a subsample by successively skipping five voxels along each of the three dimensions, and took as feature set the union of the three subsampled maps. We will also report results for the case of skipping only two voxels, rather than five.

Since high-dimensional nonlinear registration (warping) of all individuals to a common atlas (via HAMMER [33]) is applied in producing our features, they capture both volumetric and morphometric brain characteristics, which is important since individuals with AD/MCI typically exhibit brain atrophy (affecting both volume and shape).

### 2.3 Classification and feature selection for high-dimensional images

A challenge in building classifiers for medical images is the relative paucity of available training samples, compared to the huge dimensionality of the voxel space and, thus, to the number of parameters in the classifier model – in general, the number of parameters may grow at least linearly with dimensionality. In the case of 3D images, this could imply even millions of parameter values (e.g. one per voxel) need to be determined, based on a training set of only a few hundred patient examples. In such cases, classifier overfitting is likely, which can degrade generalization (test set) accuracy. Here we will apply a linear discriminant function (LDF) classifier with a built-in mechanism to avoid overfitting and with design complexity that scales well with increasing dimensionality - the support vector machine (SVM) [34]. The choice of LDF achieving perfect separation (no classification errors) for a given two-class training set is not unique. The SVM, however, is the *unique* separating LDF that maximizes the *margin*, *i.e.* the minimum distance to the classifier decision boundary, over all training samples. In this sense, the SVM maximizes separation of the two classes. For an SVM, unlike a standard LDF, the number of model parameters is bounded by the number of training samples, rather than being controlled by the feature dimensionality. Since the number of samples is the much smaller number for medical image domains, in this way the SVM greatly mitigates overfitting. SVMs have achieved excellent classification accuracy for numerous scientific and engineering domains, including medical image analysis, [26], [4], [27].

Even though SVMs are effective at mitigating overfitting, generalization accuracy may still be improved in some cases by removing features that contribute little discrimination power. Moreover, even if generalization accuracy monotonically improves with increasing feature dimensionality, high complexity (both computation and memory storage) of both classifier design and class decisionmaking may outweigh small gains in accuracy achieved by using a huge number of features. Most importantly here, it is often useful to identify the critical subset of features necessary for achieving accurate classification – these “markers” may shed light on the underlying disease mechanism. In our case, this will help to identify prognostic brain regions, associated with MCI conversion.

Unfortunately, there is a huge number of possible feature subsets, with exhaustive subset evaluation practically prohibited even for a modest number of features, 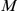, let alone 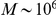. Practical feature selection techniques are thus heuristic, with a large range of tradeoffs between accuracy and complexity [35]. “Front-end” (or “filtering”) methods select features prior to classifier training, based on evaluation of discrimination power for individual features or small feature groups. “Wrapper” methods are generally more reliable, interspersing sequential feature selection and classifier design steps, with features sequentially selected to maximize the current subset's joint discrimination power. There are also embedded feature selection methods, e.g. for SVMs, use of 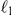-regularization within the SVM design optimization [36], in order to find “sparse” weight vector solutions, which effectively eliminate many features. For wrappers, there is greedy forward selection, with “informative” features added, backward elimination, which starts from the full set and removes features, and more complex bidirectional searches. In our work, due to the high feature dimension, we focus on two backward elimination wrappers that afford practical complexity: i) the widely used recursive feature elimination algorithm (RFE) [27], where at each step one removes the feature with least weight magnitude in the SVM solution. RFE has been applied before to AD [4], [5], [13]; ii) the recent margin-based feature elimination (MFE) algorithm [26], which uses the same objective function (margin) for feature elimination, one consistent with good generalization, that the SVM uses for classifier training [34]. MFE was shown in [26] to outperform RFE [27] and to achieve results comparable to embedded feature selection for domains with up to 8,000 features (gene microarray classification). Here we will also find that MFE gives better results than RFE.

### 2.4 An MRI-Derived Alternative to CDR-based MCI-to-AD Conversion

In the Introduction, we outlined our two classifier design steps for building an automatic prognosticator for an individual with MCI. In this section, we elaborate on these two steps and give an illustrative example. Our AD-Control classifier, used in the first step, is discussed in Sec. 2.4.1, and our second classifier, used to discriminate converter-by-trajectory (CT) and nonconverter-by-trajectory (NT) classes, is discussed in Sec. 2.4.2.

#### 2.4.1 AD-Control classifier

For the AD training population, we chose individual AD visit images with a CDR score of at least 1. For the Control training population, on the other hand, we only chose *initial* visits, and only those for participants who stayed at CDR = 0 throughout all their visits. Thus, we excluded Controls with “questionable dementia” (i.e., CDR = 0.5) at any visit. By these choices, we sought to exclude outlier examples or even possibly any mislabeled examples, recalling that CDR for the majority of both AD and MCI participants is 0.5 throughout all visits.

#### 2.4.2 CT-NT classifier

**Figure 1:**
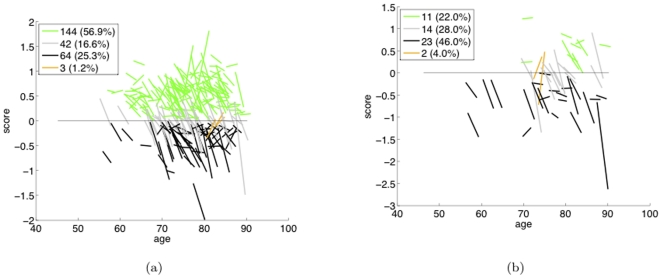
AD-Control SVM score trajectories for MCI subjects. (a) Nonconverters-by-CDR. (b) Converters-by-CDR.

Fig. 1 gives an illustrative example of the phenotypical score trajectories for MCI subjects, as described in the Introduction. A positive score is on the Control side and a negative score is on the AD side – the x-axis represents the AD-Control SVM's decision boundary. Score vs. age is plotted, with each line segment a trajectory obtained by linearly fitting an individual's phenotype scores (and linearly extrapolating if there are missing visits). Nonconverters-by-CDR (N-CDR) and converters-by-CDR (C-CDR) are illustrated in (a) and (b), respectively. Green and black subjects are those whose fitted trajectory stayed on the Control side and AD side, respectively, whereas gray lines are subjects who crossed to the AD side. Thus, by our conversion-by-trajectory definition, the green group is the nonconverters-by-trajectory, and the black and gray groups together are the converters-by-trajectory. Subject counts for these groups are given in the figure legends. The outlier subjects are shown in orange – there are five, making up less than 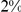 of the MCI cohort. Notice, intriguingly, from the left figure that more than one third of all (non-orange) MCI patients (106 of 298) are converters by trajectory and yet nonconverters according to CDR – *i.e.*, there is a very large percentage of patients for which the two converter definitions disagree, with the neuroimage-based definition indicating disease state changes that are not predicted using the clinical, CDR-based definition. Likewise, an additional 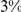 of all MCI subjects (11 of 298) “defy” their by-CDR converter label in that they do not reach the AD side of the decision boundary.

Based on these trajectories, i.e. whether or not the AD side is visited, we derive the ground-truth ‘CT’ and ‘NT’ labels for all MCI subjects. We then build a CT-NT classifier using as input *only* the image scans at initial visit. (For a small percentage of the MCI subjects, we did not obtain the patient's first visit. However, we did ensure that the visit we took as the “initial visit” had a CDR of 0.5.)

Before continuing, we note that the reader may reasonably wonder why, rather than our converter definition and associated two-stage classifier design, we do not simply directly apply the Control-AD classifier to an MCI's baseline visit image, and declare the patient a converter if the image is classified “AD” and a nonconverter, otherwise. There are several answers to this query. First, there is no longitudinal ground-truth for MCI subjects that can be derived for this alternative converter definition. Thus, one cannot evaluate this definition with respect to Desideratum 2 (generalization accuracy). Second, recall that our objective is 3-year ahead *prognosis*, not diagnosis. If nearly all of the MCI converters do actually convert *at first visit*, then our by-trajectory conversion definition would in practice be equivalent to this simpler, alternative definition. We have investigated this: from a population of 284 MCIs, there are 159 MCI converters-by-trajectory. Of these 159, 105 do in fact convert at first visit (an AD-Control classifier classifies the baseline visit as “AD”). But this means that more than one third of MCI converters-by-trajectory convert *after* the first visit, and these converters would be missed by the simpler definition. Essentially, the difference “in practice” between our proposed definition, and one which simply directly applies the Control-AD classifier to the baseline visit is quite a few more (more than one third of the total) by-trajectory converters.

## Results and Discussion

### 3.1 Introductory overview

In this section we will perform 1) classification experiments to evaluate conversion-by-trajectory and conversion-by-CDR with respect to desideratum 2; 2) additional experiments to compare the two definitions with respect to desideratum 3; and lastly, 3) experiments to identify prognostic brain “biomarker” regions.

It is important to mitigate the potential confounding effect of the subject's age. In our classification experiments, we mitigated in two ways:

1. For every classifier training, each training sample in one class was uniquely paired via “age-matching” with a training sample in the other class (with age separation at most one year).
2. For every linear-kernel SVM classifier, we separately adjusted each feature for age prior to classification using linear fitting. We subtracted the extrapolated line (computed only using ‘control” samples – for the AD-Control classifier, these are the samples in the Control class and for the CT-NT classifier we computed the line using only the NT samples) from the feature's value, for all (training and test) samples. As an aside, we note that, given the subsequent linear SVM operation, this linear fitting step is essentially equivalent to simply treating age as an additional feature input to the linear SVM classifier.

Finally, prior to building classifiers, we normalized feature values to the [0,1] range, which is suitable for the LIBSVM software [37] we used for training SVMs.

### 3.2 Experiments with voxel-based features

The test set (generalization) accuracy of the voxel-based AD-Control classifier, built using 70 training samples per class, was 0.89 (86 of 88 Controls, and 16 of 27 AD subjects, were correctly classified.) This classifier, with high specificity for Controls, was then applied to a population of MCI subjects to determine the CT and NT subgroups.

#### 3.2.1 Classification experiments for the MCI population

**Figure 2:**
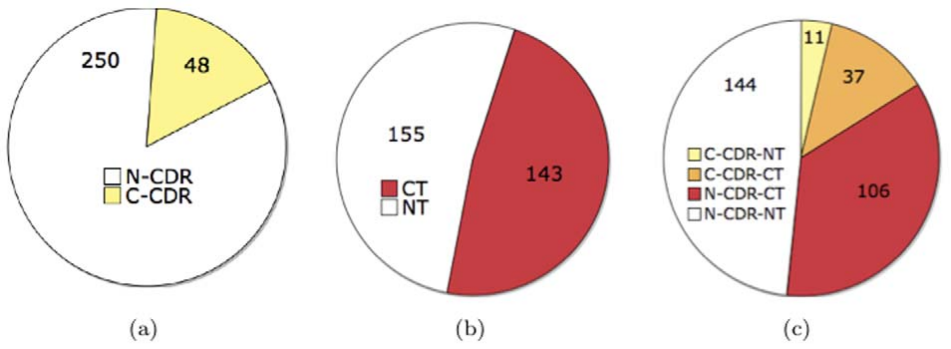
A population of 298 MCI subjects in ADNI is shown here, broken up according to the two criteria discussed in Sec. 3.2.1: (a) by-CDR criterion, (b) by-trajectory criterion; (c) overlap shown.

Fig. 2(a) shows the sizes of the converter-by-CDR (C-CDR) and nonconverter-by-CDR (N-CDR) groups within the ADNI MCI cohort for a typical experiment in our work. Fig. 2(b) shows the same population broken up as converters-by-trajectory (CT) and nonconverters-by-trajectory (NT). Superimposing the two charts, Fig. 2(c) illustrates their overlap, where converters by both definitions are accordingly indicated by orange. Since converters-by-CDR are relatively scarce, we used a large majority of them (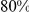, i.e. 39 individuals among the 48) for the by-CDR classifier's training set, with the rest (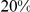) put into the test set. We reiterate that a general disadvantage of the by-CDR approach is its scarcity of converter examples – by contrast, a more balanced number of examples is available for by-trajectory training (at least 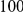, rather than 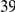, training samples per class, as in Fig. 2(b)). Note also that if we were to use a “probable AD”, rather than a by-CDR converter definition, where converters are required to have undergone both CDR and MMSE changes, there would necessarily be even *fewer* converter examples, which makes “probable AD” even less attractive than by-CDR from the standpoint of having an adequate sample for classifier training and testing. This also raises the possibility of an *alternative* clinically-based definition, based on a logical OR-ing of CDR-based and MMSE-based conversions, *i.e.* where a converter must *either* have undergone CDR change or MMSE changes (or both). One difficulty here is how to define MMSE-based conversion. In [13] MMSE scores were averaged over all visits in order to reduce noise. One can accordingly then define MMSE-based conversion if a subject's MMSE score, averaged over all visits, falls below a given threshold. The average MMSE score over *all* MCI subjects is 25.85. To assess the number of additional cognitive score-based converters one could obtain by considering MMSE, we varied a threshold on the average MMSE score, evaluating at cutoffs of 24, 23, and 22, and finding that the additional number of converters declared in this way were 55, 35, and 22, respectively. Thus, especially for an MMSE cutoff of 24, one can obtain a significant number of extra converters using MMSE in addition to CDR. However, it is unclear what is in fact a proper choice for the MMSE threshold – simply choosing a threshold at 24 because this leads to more converters is somewhat arbitrary, without a strong objective basis. Accordingly, in the sequel, for evaluating clinically-based conversion, we will only experimentally evaluate by-CDR conversion, as used in [4], [5], [13]. Specification of a principled combined CDR and MMSE-based conversion definition and validation of such a definition is a good subject for future work.

A fair performance comparison between by-trajectory and by-CDR classification requires: 1) using the same per-class training set size (i.e. 39) for both by-CDR and by-trajectory training, *and* 2) making the test set sizes the same for both classifiers. There are several different ways in which the data can be partitioned into training and test sets, consistent with these two conditions: i) we can perform simple random selection on a class-by-class basis, ensuring only that the two classifiers are given the same training/test set *sizes* (but not the same *sets*) – note that this means that the training sets for the converter and nonconverter classes of the conversion-by-CDR classifier are randomly selected from the yellow and white regions in Fig. 2(a), respectively, with *no* consideration of trajectory-based (i.e. red/white) labeling illustrated in Fig. 2(b); ii) we can make the training sets of the two classifiers *identical* rather than *merely same-sized*, as well as make the test sets identical. This latter approach, though, will have some bias because, in selecting samples for the by-trajectory classifier, we will have to make use of knowledge of the samples' conversion-by-CDR status (and vice versa for the by-CDR classifier). The first approach, on the other hand, clearly does not have this bias. As both approaches are valid ways of dealing with by-CDR data limitations, we will compare generalization accuracies of by-CDR and by-trajectory classifiers under both these data selection schemes, respectively, referring to these approaches as “random” and “identical” in the sequel.

**Figure 3:**
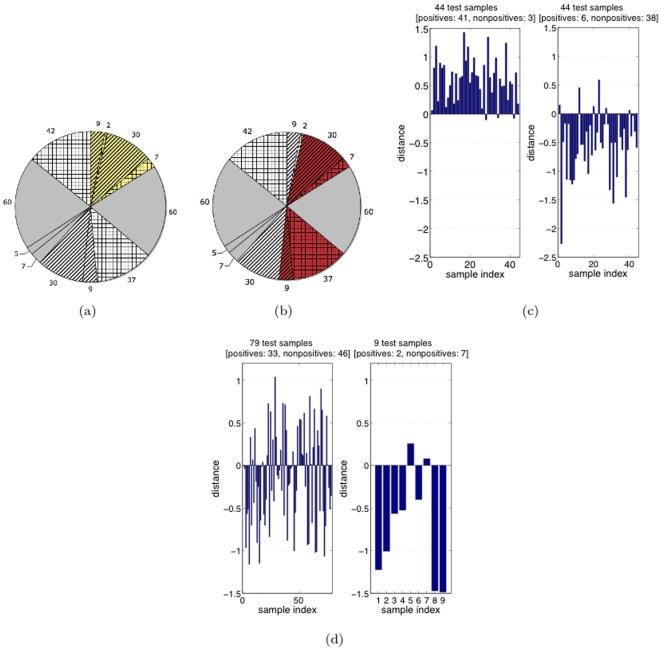
Test set accuracy comparison of by-CDR and by-trajectory classification. (a) Training/test set selection for by-CDR classification. (b) Training/test set selection for by-trajectory classification. (c) By-trajectory. Left: nonconverters; Right: converters. (d) By-CDR. Left: nonconverters; Right: converters.

Our training/test set selection procedure for the “identical approach” is as follows. For the C-CDR-CT group (Fig. 2(c)), randomly select 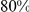 of the group (the yellow striped group of size 30 in Fig. 3(a)) such that a corresponding group within N-CDR (white portion in Fig. 2(a)) can be found that is both *NT* and satisfies age-matching. This corresponding group is illustrated in Fig. 3(a) as the white striped group (of size 30), placed opposite from the yellow striped area it is paired (matched) with. Likewise for the C-CDR-NT group (Fig. 2(c)), randomly select 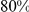 of the group (the yellow striped group of size 9 in Fig. 3(a) such that a corresponding group within N-CDR can be found that is both *CT* and satisfies age-matching. This corresponding group is illustrated in Fig. 3(a) as the white striped group (of size 9). Notice by comparing this figure to Fig. 2(c) that the two white striped areas are separated by the CT-NT border. We take the training set – shared by the by-CDR and by-trajectory classifiers – to be precisely the union of these four striped areas. (For the by-CDR classifier, the class membership of any of these four subsets of the training set is illustrated by the color being yellow or white in Fig. 3(a). Likewise, for the by-trajectory classifier, class membership is illustrated by red or white color in Fig. 3(b).) Subsequently, we take the test set – shared by the two classifiers – to be the subjects who are neither in 1) the training set (striped areas) nor in 2) the special set of subjects shown in solid gray in Fig. 3(a) (also shown identically in Fig. 3(b)). We exclude this “special set” (in gray) from the *test set* so that *all* our experiments under the “identical approach” can have a shared, fixed test set (for fair comparison with each other), including, crucially, an experiment that will include this “special set” of samples in the *training set*. That is, the test set is the tiled areas in Fig. 3(a) (or, identically, in Fig. 3(b)).

Note above that *some* random selection is being employed in choosing the training/test sets even in the “identical approach” (whereas, in the “random approach” the selection is completely random). Thus, for both approaches, the accuracy of performance comparison will benefit from averaging accuracy results over multiple training/test split “trials”, where the training and test sets will vary from trial to trial based on the random selection that is built into the data selection procedure (for both the “random” and “identical” approaches). This essentially amounts to a bootstrap procedure, which aims to work with a finite (limited) amount of data and, at the same time, both build accurate models and accurately *assess* the model's generalization accuracy [38]. Results averaged across 10 trials are given in Table 1 for a linear-kernel SVM (for generating all classification results herein, including those in Table 1, we used SVM classifiers that were built by employing the common approach of bootstrap-based validation for selecting the classifier's (trial's) hyperparameter values [26]); 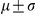 notation is used to indicate the mean 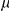 and standard deviation 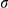 of quantities across the trials, which are shown rounded up. Note that by-trajectory's generalization performance is as high as 0.83, whereas by-CDR's generalization performance is very poor – *as poor as random guessing* (see 0.5 and 0.56 table values) – due mainly to poor performance on *nonconverters*-by-CDR. Fig. 3(c) and Fig. 3(d) show by-trajectory and by-CDR results, respectively, for one of the 10 trials (for the “random approach”), with each bar indicating distance to the classification boundary for an MCI subject in a test population of size 88 and nonconverters/converters shown in left/right figures, respectively. Positive/negative distance means nonconverter/converter side of the boundary, respectively. Among the 88 subjects, by-trajectory correctly classified 79 whereas by-CDR correctly classified only 40.

**Table 1:** Test set accuracy comparison of by-CDR and by-trajectory classification: Average test set classification accuracy using all 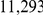 features.

Sample	Classifier	Test set				
selection		Converters		Nonconverters		Overall
		Count	Accuracy	Count	Accuracy	accuracy
**Random**	By-trajectory	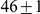	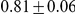	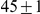	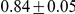	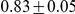
**approach**	By-CDR	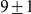	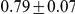	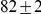	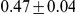	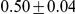
**Identical**	By-trajectory	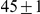	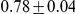	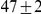	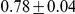	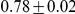
**approach**	By-CDR	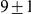	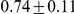	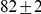	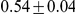	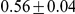

Recently, similarly poor by-CDR classification performance was also reported in [4], where it was found that the majority of (by-CDR) nonconverters “had sharply positive SPARE-AD scores indicating significant atrophy similar to AD patients”. Since the SPARE-AD score is produced by a classifier that was trained to discriminate Control and AD patients [32], [16], this comment and associated results are consistent both with our conjecture in the Introduction and our above histogram results, which suggest that there may be a significant number of patients undergoing physiological brain changes consistent with conversion, yet without clinical manifestation.

The results above indicate that the conversion-by-CDR definition's two classes are not well-discriminated, and thus, clinical usefulness of this definition for our prognostic Aim is expected to be poor. The much greater generalization accuracy of the by-trajectory definition (coupled with its inherent plausibility as a conversion definition) indicates its greater utility.

*Increasing the By-Trajectory Image-Based Feature Resolution:* In a separate experiment, we evaluated using one of 27 subsamples (rather than one of 216 subsamples), i.e. a 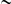
10-fold increase in the number of (voxel-based) features, and found that the by-trajectory generalization accuracy rose to 0.91 in the “random” case. We then tried building 27 separate by-converter classifiers, one for *every* 1/27th subsample (thus effectively using the whole 3D image), with majority-based voting used to combine the 27 decisions. This ensemble scheme again achieved 0.91 accuracy, i.e. there was no further accuracy benefit beyond that from a 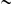
10-fold increase in the number of voxel features.

**Figure 4:**
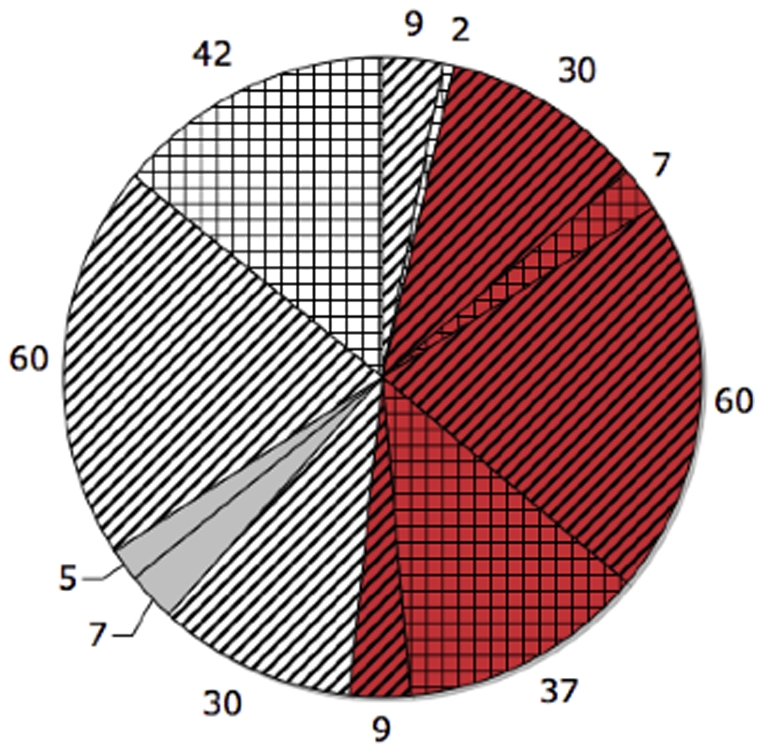
Training/test set selection for by-trajectory, considering a larger training set size (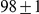 per class) and 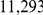 features.

*Increasing the By-Trajectory Training Set Size:* Note that the converter-by-CDR sample scarcity and class-balancing (via age-matching) in the experiments above had the effect of artificially limiting the *by-trajectory* classifier training set size. Next we investigated how much the generalization accuracy of by-trajectory classification improves when this limitation is removed. The tiled areas in Figures 3(a) and 3(b) are identical, illustrating that in this new experiment (Fig. 4 and Table 2) we used the same test set as previously, for fairness of comparison. However, as indicated by differences in the total striped area between these two charts, we now make the training set much larger than previously. Specifically, for the “identical” case, we used the previous 10 trials but simply augmented a trial's training set with the two large, previously-excluded gray sets, shown in Fig. 3(b), with size 60, as these two sets do age-match each other. The results, averaged across the 10 trials, are given in Table 2. Notice in this figure the now larger per-class training size (on average 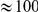 rather than 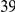), and that the random approach uses this size as well. The by-trajectory results in Table 2 indicate that accuracy improved from 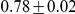 (Table 1) to 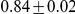 for the “identical approach”, and modestly worsened for the “random approach” (from 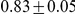 to 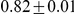).

**Table 2:** By-trajectory average test set classification accuracy for the larger training set size (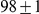 per class) and 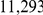 features.

	Sample	Test set				
	selection	Converters		Nonconverters		Overall
		Count	Accuracy	Count	Accuracy	accuracy
**By-trajectory**	**Random**	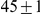	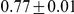	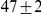	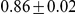	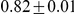
	**Identical**	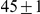	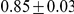	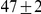	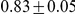	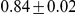

*A Definition Based on Both Neuroimaging and Clinical Change:* We now consider a third definition of conversion that combines the first two definitions as follows. Let “converters” consist of individuals who converted either by-trajectory *or* by-CDR (non-white areas in Fig. 2(c)), with the “nonconverter” class consisting of the remaining MCI individuals (white area). We give two points of view on this new definition, “conversion-by-union”. First, despite the disadvantages with CDR and MMSE pointed out in sections 1 and 2 of this paper, our strictly neuroimaging-based by-trajectory definition is not using any clinical information in defining the phenotype, *i.e.* psychological effects are not being taken into account. Second, this “union” definition is more *inclusive* in defining an MCI subpopulation at risk, which may benefit from early treatment or diagnostic testing. While from that perspective the new definition is reasonable, the fact that grouping individuals by CDR has a role in this definition may be its disadvantage, considering that by-CDR classification was previously shown to perform not much better than random guessing. Results, averaged across the same 10 trials used in Fig. 3, are given in Table 3 and indicate that conversion-by-union generalizes somewhat worse than conversion-by-trajectory. Note that “by-union” is, by definition, an instance of the “identical approach”. To ensure fairness of comparison with the by-trajectory definition of conversion, our test sets, and training set sizes, in these two cases were identical. In fact, we chose the by-union training set to be as similar to by-trajectory's, in every trial, as possible. Referring to Fig. 3(b) (which represents a trial example), the by-union training set was chosen to include 1) the two large striped groups (red and white); 2) the small “special” gray group (of size seven in this trial example) and its age-matched counterpart within small white-striped group, and; 3) a subset of the second small “special” gray group (two of five individuals in this trial example) and its age-matched counterpart within the small red-striped group. We do not further evaluate “conversion-by-union” here. We do, however, identify “optimal” definition of a multidimensional phenotype and associated conversion, based on neuroimaging, multiple cognitive measures ( *e.g.*, CDR and MMSE), genetic markers, and CSF markers (if routinely measured) as a good direction for future work.

**Table 3:** Average test set accuracy of by-union classification for 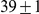 per-class training samples and 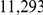 features.

	Sample	Test set				
	selection	Converters		Nonconverters		Overall
		Count	Accuracy	Count	Accuracy	accuracy
**By-union**	**Identical**	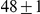	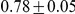	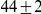	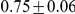	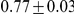

#### 3.2.2 Validation on Known AD Conversion Biomarkers

To validate the proposed conversion definitions with respect to desideratum 3, we performed correlation tests on the MCI population between the binary class variable 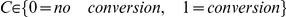 and known AD conversion biomarkers consisting of: 1) volume in reported AD-affected regions (Table 2 in [11]), which we measured for each individual's *final-visit* MRI (As discussed in the supplemental Document S1, we measured *normalized* region volume. Note also that our regions are defined based on the atlas (Atlas2) we used. The correspondence between the regions in [11] and our defined regions is given in Table 4. Finally, note that a subject's final visit is not always the sixth visit.); 2) the following CSF-based markers, as considered previously in [12]: tau, p-tau, A
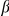
1-42; and 3) the clinical MMSE measure. The stronger the correlation, the more accurately the biomarker is predicted from the class variable and the greater the separation between the biomarker histograms, conditioned on the two classes. In particular, we would expect that a good converter definition should have statistically significant correlation between its class variable and region volume at final visit for known marker regions such as the hippocampus. We note, however, that for measuring correlation between the by-trajectory class label 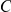 and the final-visit MRI-derived region volume biomarkers, some care is required to avoid statistical bias. In particular, note that the by-trajectory label is obtained by applying the Control-AD classifier to each visit's MRI, with conversion declared if any of the visits (including the final one) is classified as “AD”. Since the final visit is also used to measure the region volume biomarkers we will use to *validate* the by-trajectory labels, this “dual use” of the final visit would be a source of bias. There are 22 MCI subjects that by-trajectory convert only at the final visit. To avoid bias in validating the by-trajectory approach, we excluded these 22 subjects from brain region-volume based statistical validation of the by-trajectory definition. The full MCI population (including these 22 subjects) was used in all our other validation testing.

**Table 4:** Correspondence between the regions in [11] (left) (except “Total GM” and “Total WM”) and our defined regions (right).

Entorhinal cortex	Entorhinal cortex left/right
Fusiform gyrus	Lateral occipitotemporal gyrus right/left
Hippocampus	Hippocampal formation right/left
Inferior parietal GM	Supramarginal gyrus left/right, Angular gyrus right/left
Lateral orbitofrontal GM	Lateral front-orbital gyrus right/left
Lateral ventricles	Lateral ventricle left/right
Medial orbitofrontal GM	Medial front-orbital gyrus right/left
Parahippocampal gyrus	Parahippocampal gyrus right/left
Posterior cingulate	Cingulate region left/right
Precentral GM	Precentral gyrus right/left
Superior frontal GM	Superior frontal gyrus left/right
Superior temporal GM	Superior temporal gyrus right/left

**Figure 5:**
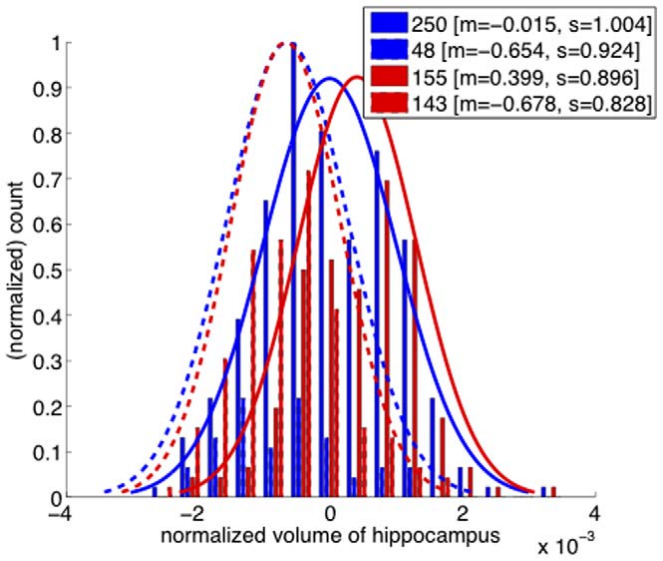
For the hippocampus, by-trajectory (red) has larger histogram separation between converter (dashed line) and nonconverter (solid line) groups than by-CDR (blue). To illustrate this more clearly, also shown is the Gaussian curve for each of these four subject groups (plotted based on group mean (m) and standard deviation (s) indicated in the figure legend with the same 0.001 scaling as the x-axis).

Before presenting correlation test results, we first illustrate in Fig. 5 the increased separation of the histograms of hippocampus volume for the converter and nonconverter groups in the by-trajectory case, compared with by-CDR. Next, we performed comprehensive statistical tests for a number of suggested AD biomarkers. The R statistical computing package was used to perform all tests with statistical significance set at the 0.05 level. In Table 5a, the correlation coefficients for by-trajectory and by-CDR are shown for each biomarker, along with their associated p-values [39]. Note that for 10 out of 14 brain regions, the correlation with by-trajectory is greater than the correlation with by-CDR (in **bold**), with by-trajectory meeting the significance threshold in 9 of these 10 regions. Further, for only two of the remaining four biomarkers - posterior cingulate and the clinical MMSE measure - does the correlation with by-CDR meet the significance threshold. Most notably, well-established markers for AD such as the hippocampus, lateral ventricles, and inferior parietal exhibited strong correlation with the by-trajectory definition. To further assess statistical significance of the *comparison* between by-trajectory and by-CDR correlations, we performed a *correlated correlation test* [40], the appropriate test given that the same MCI sample population (excepting 22 excluded subjects for the by-trajectory brain region volume tests) was used in measuring correlations for both by-CDR and by-trajectory. This test (Table 5b) reveals that the larger correlation of by-trajectory is statistically significant at the 0.05 level in six brain regions (in **bold**), most notably the hippocampus, with a very low p-value (2.16e-10). By contrast, conversion-by-CDR does not achieve a statistically significant advantage for any of the brain regions, nor with respect to MMSE.

**Table 5:** Correlation coefficients and associated p-values: (a) Correlation test results; (b) Correlated correlation test results for each of the regions in (a).

	(a)				(b)
	By-trajectory		By-CDR		
	Correlation	P-value	Correlation	P-value	P-value
Biomarker	coefficient		coefficient		
Entorhinal cortex	**0.180**	3.14E-03	0.054	0.382	0.092
Fusiform gyrus	**0.262**	1.45E-05	**0.157**	0.010	0.154
Hippocampus	**0.645**	2.00E-16	**0.245**	4.99E-05	**2.16E-10**
Inferior parietal GM	**0.249**	3.72E-05	0.097	0.112	**0.042**
Lateral orbitofrontal GM	0.064	0.300	0.113	0.064	0.507
Lateral ventricles	**0.452**	6.76E-15	**0.194**	0.001	**2.18E-04**
Medial orbitofrontal GM	0.083	0.173	**0.137**	0.025	0.476
Parahippocampal gyrus	**0.311**	2.09E-07	0.063	0.305	**7.75E-04**
Posterior cingulate	0.026	0.666	**0.164**	0.007	0.067
Precentral GM	0.055	0.372	0.089	0.145	0.647
Superior frontal GM	**0.240**	7.37E-05	0.116	0.057	0.096
Superior temporal GM	**0.325**	4.99E-08	**0.170**	0.005	**0.003**
Total GM	**0.404**	6.34E-12	**0.260**	1.60E-05	**0.039**
Total WM	0.102	0.094	0.066	0.282	0.628
MMSE	**0.343**	8.16E-09	**0.438**	5.84E-14	0.159

Statistically significant results are shown in bold.

Statistical testing results for the CSF markers are shown in Table 6. As seen in the table, by-trajectory has larger correlation with tau and A
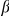
1-42 than by-CDR conversion. Moreover, for by-trajectory, these correlations are statistically significant. However, the correlated correlation test did not indicate that the *comparison* of correlations reached a statistically significant level. Correlations with p-tau were comparable for the two conversion definitions.

**Table 6:** Correlation coefficients and associated p-values: (a) Correlation test results; (b) Correlated correlation test results for each of the CSF biomarkers in (a).

	(a)				(b)
	By-trajectory		By-CDR		
	Correlation	P-value	Correlation	P-value	P-value
CSF Biomarker	coefficient		coefficient		
Tau	**0.169**	0.0457	0.119	0.159	0.624
Ptau-181	0.159	0.0604	0.153	0.0698	0.957
A 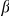 42	**0.255**	2.25E-3	0.092	0.278	0.104

Statistically significant results are shown in bold.

To summarize, testing on both brain region and CSF-based markers validates that by-trajectory is more consistent with conversion to AD than the by-CDR definition.

#### 3.2.3 Identification of prognostic brain “biomarker” regions

**Figure 6:**
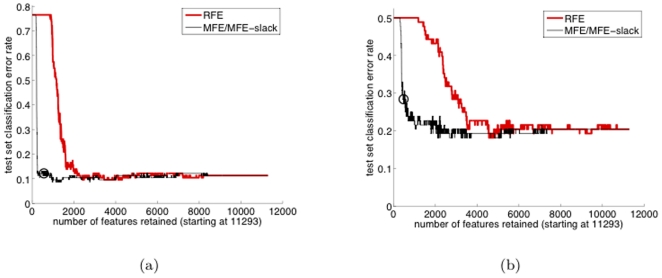
Test set misclassification rate during the course of feature elimination for: (a) the AD-Control classifier and (b) the CT-NT classifier.

In the previous section, we validated conversion definitions using established (diagnostic) AD biomarker brain regions (with volumes measured at final visit). In this section, we will *identify* key *prognostic* biomarker brain regions (from the baseline visit image) via supervised feature selection, aiming first to identify the “essential” subset of voxel features, i.e. the voxels (at initial visit) necessary for our classifier to well-discriminate the CT and NT classes. The brain regions (consistent with a registered brain atlas) within which these select voxels principally reside then identify our prognostic brain biomarker regions. Similarly, we will identify *diagnostic* regions, critical for discriminating between AD and Control subjects (using our AD-Control classifier). In both cases, the accuracy of the selected brain region biomarkers rests heavily on the accuracy of the supervised feature selection algorithm we employ. In Figure 6, we compare MFE and RFE feature elimination (i.e. feature selection via feature elimination) for both Control-AD classification and for CT-NT classification (for one representative, example trial). The curves show test set accuracy as a function of the number of retained features (which is reduced going from right to left). Note that the “MFE/MFE-slack” hybrid method [26]) outperforms RFE for both brain classification tasks, achieving lower test set error rates, and with much fewer retained features. The circle, determined without use of the test set based on the rule in [26], marks the point at which we stopped eliminating features by MFE, thus determining the (trial's) retained voxel set. This MFE-RFE comparison (and the previous comparison in [26]) supports our use of MFE to determine brain biomarkers.

**Figure 7:**
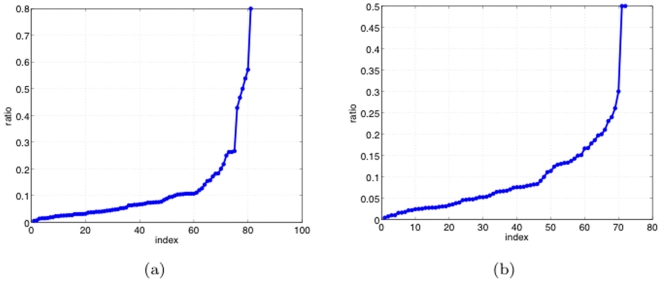
Sorted retained voxel percentages for initial regions used to select final regions (Sec. 3.2.3): (a) AD-Control; b) CT-NT.

To relate the retained voxel set to anatomic regions in the brain, we overlaid the retained voxel set onto a registered atlas space. For CT-NT classification, to improve robustness, the final voxel set was formed from the union of the retained voxel sets from each of ten feature elimination trials (each using a different, randomly selected training sample subset). For AD-Control classification, the final voxel set came from a single trial (the only trial, from which the 10 CT-NT trials stemmed). For each of these two cases, overlaying the final voxel set onto the co-registered atlas (Atlas2, defined in the supplemental Document S1) yielded between 70–80 anatomic regions. For data interpretation purposes, we then identified a subset of (biomarker) regions using the following procedure. First, for each brain region, we measured the percentage of the region's voxels that are retained, *sorted* these percentages, and then plotted them. As shown in Fig. 7(a), the resulting curve for the AD-Control case has a distinct knee, which we thus used as a threshold (0.125) to select the final, retained (diagnostic) regions for AD-Control. We used the same threshold for the CT-NT curve, shown in Fig. 7(b). This choice of threshold yields a reasonable number of regions – 19 for the CT-NT (prognostic) case and 21 for the AD-Control (diagnostic) case.

The resulting sets of identified prognostic and diagnostic biomarkers are given in Table 7, along with their intersection. The diagnostic markers in the table include the majority of the known brain regions in the medial temporal lobe involved in AD pathology. For example, hippocampus atrophy and lateral ventricle enlargement, particularly in its anterior aspects of the temporal horn, are considered the most prominent diagnostic markers for AD. Entorhinal cortical regions, including the perirhinal cortex, are presumably the earliest sites of degeneration [41]. Thus, independent identification by our AD-control classifier of known AD diagnostic biomarkers establishes a reasonable basis for applying the same approach to identify prognostic biomarkers. The brain regions listed as CT-NT prognostic markers include most known AD diagnostic markers (including 8 of the 12 regions from [11] (marked by *), 4 of which are also diagnostic markers), indicating that some AD-linked pathological changes in these brain regions already occurred and remained active in a subset of MCI subjects who likely progress to AD rapidly. Conversely, the brain areas appearing only on the prognostic marker list are likely the most active areas of degeneration during this stage of progression to dementia. These structures tend to be the brain regions further away from the entorhinal cortex onto the parietal (Supramarginal gyrus, Precuneus) and temporal cortex (Superior temporal gyrus and Middle temporal gyrus) regions. All the brain structures listed in the table are known to be involved in AD [41], [42], [43]. Thus, the markers in Table 7 suggest an interesting anatomic pattern of trajectory for MCI conversion to AD which conforms with the Brak and Brak hypothesis and previous imaging findings [42], [43]. Moreover, the CT-NT regions uniquely found by our MFE-based procedure in Table 7 may be viewed as “putative” prognostic markers, and may warrant further investigation.

**Table 7:** Brain regions identified as biomarkers using voxel-based features and MFE.

AD-Control classifier only	intersection	CT-NT classifier only
Amygdala left	Hippocampal formation* left	Superior temporal gyrus* left
Cingulate region right	Hippocampal formation* right	Middle temporal gyrus left
Entorhinal cortex right	Entorhinal cortex* left	Precuneus right
Inferior occipital gyrus right	Inferior temporal gyrus right	Lateral front-orbital gyrus* right
Medial occipitotemporal gyrus left	Lateral occipitotemporal gyrus* right	Insula right
Parahippocampal gyrus left	Parahippocampal gyrus* right	Supramarginal gyrus* left
Temporal lobe WM right	Perirhinal cortex left	Temporal lobe WM left
Temporal pole right	Perirhinal cortex right	Temporal pole left
	Middle temporal gyrus right	Medial front-orbital gyrus* left
	Uncus left	

Finally, we note that we have used a particular criterion (percentage of a region's voxels that are retained) to identify biomarker regions, starting from MFE-retained voxels. While our identified regions are plausible, it is possible that other (equally plausible) criteria may produce different biomarker region results. Thus, the biomarkers we identify should be viewed as anecdotal, identifying regions that figure prominently in our classifier's decisionmaking and also potentially assisting researchers in forming hypotheses about MCI-to-AD disease progression. However, we do not view the identified regions as definitive.

#### 3.2.4 Comparison with an SPM-based biomarker identification approach

In the previous section, we used MFE to identify voxels as biomarkers for the CT and NT classes. Here, using the same CT-NT training and test populations, we will alternatively identify voxel-based biomarkers using statistical testing with SPM5 (see: [44]). Subsequently we will present a classifier generalization accuracy comparison (where accuracy is again measured on the previous section's CT-NT (*test*) population) for these two biomarker detection methods. We determined SPM biomarkers as follows. The CT-NT training set population, being age-matched, is readily suitable for a paired t-test, an appropriate statistical test for determining SPM-identified biomarkers, i.e. voxels that discriminate between the CT and NT groups. In contrast with MFE's use of *only one* (out of 216) RAVENS subsamples (taken jointly from the GM, WM, ventricle maps), we performed t-tests on *whole* RAVENS maps (without subsampling), which makes the SPM-MFE comparison favorably biased towards SPM. More specifically our steps were as follows. First, for the GM and WM maps separately, we found using SPM that a large portion of each of these two tissues was statistically significant at the 0.05 level when correction for multiple comparisons was not applied. Next, we used SPM's FDR-based correction for multiple comparisons – based on an SPM FDR cluster size of 5 voxels we found that the spatial extent of the statistically significant regions, at each of the levels 0.05, 0.01, and 0.005, was approximately a subset of the above-mentioned spatial support found in the uncorrected case. Given that the number of significant voxels in any of these SPM experiments is, again due to no subsampling, much larger than the 11,293 voxels started from in the MFE case, we simply 1) chose as our SPM result the result for 0.01 (FDR-corrected), 2) took from among those significant voxels the *most* significant 11,434 voxels in order to be able to compare MFE and SPM for
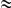
the same number of voxels (biomarkers). To obtain the generalization accuracy for this SPM-identified biomarker voxel set, using the same training/test set as in the MFE experiment, we trained an SVM classifier and measured its generalization accuracy, which was found to be 0.76. This accuracy is somewhat lower than the 0.8 accuracy of the previous section's CT-NT SVM classifier. Recalling that this comparison is actually favorably biased towards SPM, and further noticing the fact that MFE was able to maintain the 0.8 accuracy all the way down to 2000 features (cf. Fig. 6(b)), this experimental comparison provides another validation (beyond the comparison with RFE given earlier) for MFE-based feature/biomarker selection, applied to brain images.

### Conclusions

We have presented an automated prognosticator of MCI-to-AD conversion based on brain morphometry derived from high resolution ADNI MR images. The primary novel contributions of our work are: i) casting MCI prognostication as a novel machine learning problem lying somewhere between supervised and unsupervised learning; ii) our proposal of a conversion definition which, unlike previous methods, exploits both rich phenotypical information in neuroimages and AD and control examples; iii) correlation testing and classifier accuracy evaluations to validate candidate conversion definitions; iv) prognostic brain region biomarker discovery based on our conversion definition. We demonstrated that our method achieved both better generalization accuracy and stronger, statistically significant, correlations with known brain region biomarkers than a predictor based on the clinical CDR score, the approach used in several past works. Our method also achieved higher correlation with CSF markers than CDR-based conversion. The brain structures identified as AD-control diagnostic markers and MCI conversion prognostic markers well conform with known brain atrophic patterns and progression trajectories occurring in AD-afflicted brains. While the noisy nature of cognitive assessments, including MMSE, has been acknowledged in past works, in future, in order to exploit all relevant information sources, we will aim to extend our methodology to consider multiple cognitive assessment measures, both potentially as additional (baseline) input features and as additional phenotypical prediction targets to our “conversion-by-trajectory” labels. We may also consider alternative ways to adjust for confounding effects of age, noting that [11] has characterized the nonlinear dependence of age on brain region volumes. While we have focused on the MCI subpopulation here, our system could also potentially be used to detect, as possible misdiagnoses, subjects diagnosed as “Control” who are classified as MCI converters by our system. Finally, we may consider the important, *allied* problem of Control-to-MCI prognostication.

## Supporting Information

Document S1 **Image Processing.**
(PDF)
